# Dermoscopy of Lip Lichen Planus—A Descriptive Study

**DOI:** 10.5826/dpc.1004a76

**Published:** 2020-10-26

**Authors:** Shekhar Neema, Sunmeet Sandhu, A.W. Kashif, Preema Sinha, Rohit Kothari, S. Radhakrishnan

**Affiliations:** 1 Department of Dermatology, Armed Forces Medical College, Pune, India; 2 Department of Pathology, Armed Forces Medical College, Pune, India

**Keywords:** dermoscopy, lip lichen planus, leaf venation-like, Wickham striae

## Introduction

Isolated lip lichen planus (LP) can mimic lip discoid lupus erythematosus, actinic cheilitis, pemphigus vulgaris, exfoliative cheilitis, and herpes simplex [1]. Dermoscopic features of cutaneous LP have been well-described in the literature, with Wickham striae (WS) as the distinctive feature of an active disease. However, the dermoscopic patterns of lip LP remain to be elucidated. Only a few case reports have reported the dermoscopic patterns of lip LP that include WS: diffuse scaling and violaceous background [1,2]. Herein, we aim to provide new insights into the dermoscopic profile of biopsy-proven cases of lip LP.

## Case Presentation

**Figure 1:**
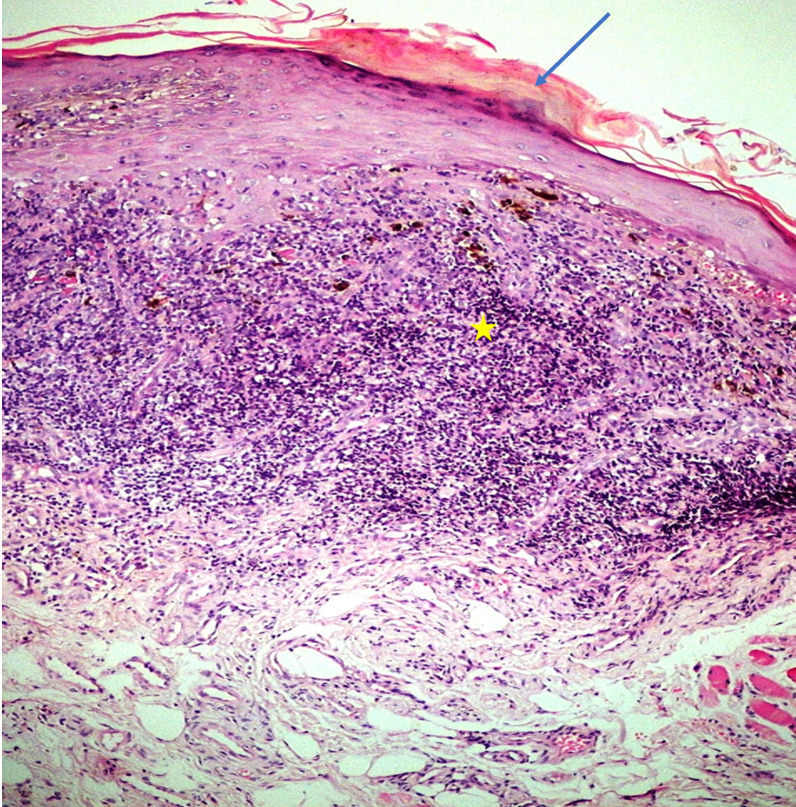
Biopsy from lip shows focal parakeratosis, hypergranulosis (blue arrow), interface dermatitis (yellow star), Civatte bodies, and pigment incontinence, suggestive of lichen planus (H&E, ×100).

A total of 12 biopsy-proven patients of lip LP who had not taken any treatment were included in the study (Figure 1). Clinical profiles of the patients are listed in Table 1. Nine cases (75%) had isolated lip involvement. One case (8.3%) had also involvement of buccal mucosa and 2 (16.6%) had additional buccal mucosal and cutaneous LP lesions. Ten patients (83.3%) had a lower lip LP. Dermoscopic images were captured using a DermLite DL4 dermatoscope attached to Samsung Galaxy Note 10 mobile phone. The images were independently analyzed by 2 expert dermatologists. Various dermoscopic features seen were WS (100%), scaling (100%), black/gray/brown/pigmentation as dots or globules (100%), vascular pattern (91.7%), erosion with bleeding spots (50%), and rosettes (41.7%) over an erythematous-to-violaceous background in all 12 cases (Table 2).

**Figure 2:**
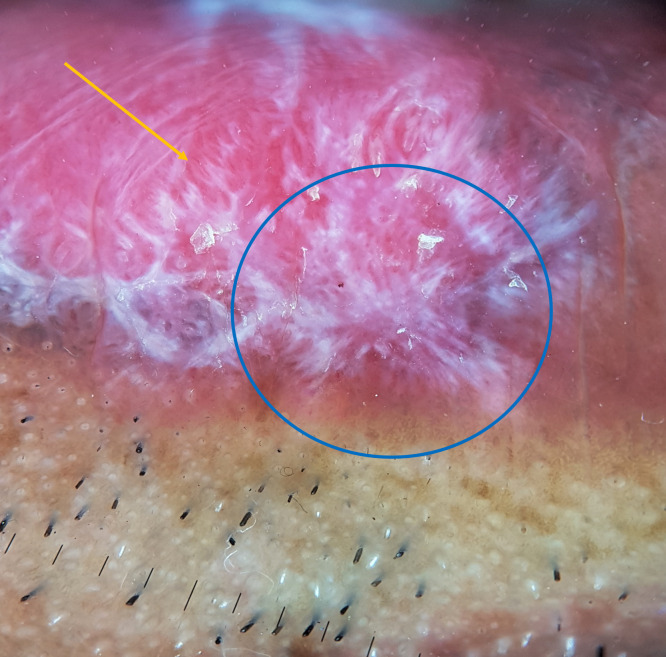
Radial pattern of Wickham striae (blue circle), erosion and hairpin vessels (yellow arrow) (DermLite DL4; polarized, ×10).

**Figure 3:**
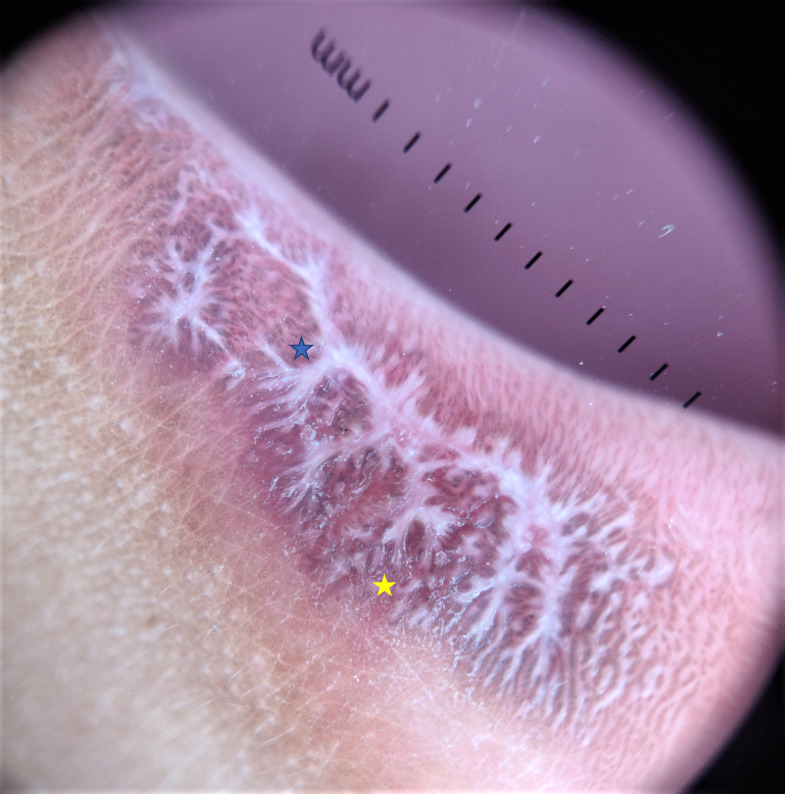
Leaf venation-like Wickham striae (blue star), scaling, dotted vessels and pigmented granules over a violaceous background (yellow star) (DermLite DL4; polarized, ×10).

**Figure 4:**
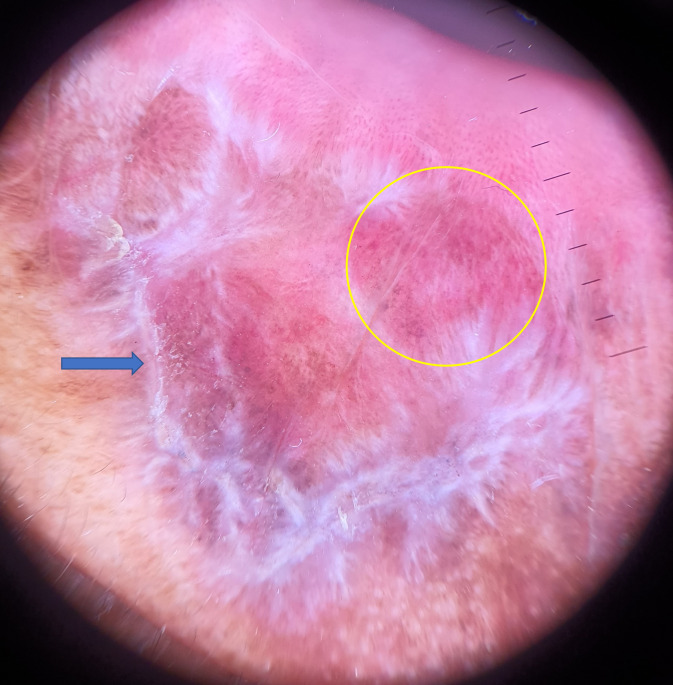
Linear Wickham striae (blue arrow), dotted, linear and hairpin vessels (yellow circle) (DermLite DL4; polarized, ×10).

**Figure 5:**
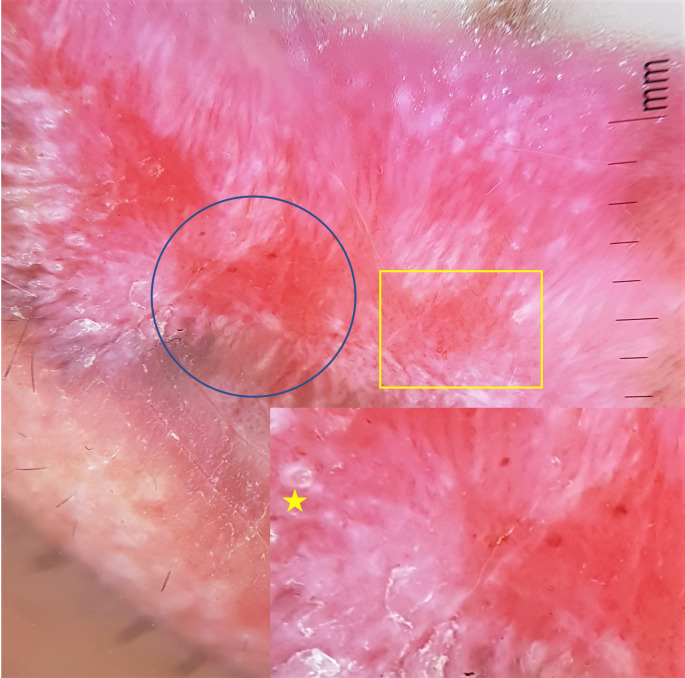
Erosion with bleeding spots and dotted vessels (blue circle), prominent hairpin and linear vessels (yellow rectangle). Inset: rosettes (yellow star), bleeding spots and hairpin vessels (DermLite DL4; polarized, ×10).

**Figure 6:**
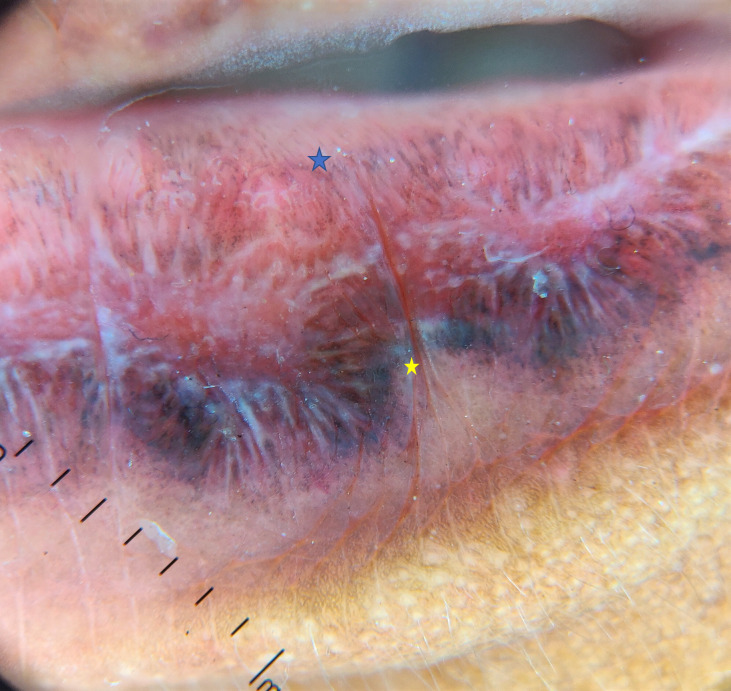
Prominent uniform-to-granular black pigmentation (yellow star) along with radial and linear Wickham striae. Gray dots and globules (blue star) (DermLite DL4; polarized, ×10).

A mixed pattern of WS and predominant radial pattern in 9/12 cases (75%) (Figure 2) was noticed. These patients had a disease duration of >9 months. A “leaf venation-like pattern” was seen in 2 cases (16.7 %) with duration of lesions of <6 months (Figure 3). Linear WS were present in 4 cases (33.3%) (Figure 4). All 6 cases (50%) with erosions had prominent hairpin and linear telangiectasia (Figure 5). Another vascular pattern seen in 9 (75%) cases was dotted (Figure 4). Black to gray-black pigmentation was highlighted in cases with disease duration of >12 months (Figure 6). On polarized dermoscopy, rosettes are seen as 4 white dots arranged in a square resembling a 4-leaf clover, which corresponds to concentric horny material in follicular and eccrine ducts at the infundibular level or peri-follicular concentric fibrosis. Interestingly this pattern was seen in 4 patients (33.3%) with prominent scaling that might correspond to polarized keratin-filled eccrine duct narrowing at the lip margin (Figure 5).

The limitation of our study was a small number of patients and the lack of a control group; therefore, the accuracy of various diagnostic criteria could not be performed.

## Conclusions

Our study provides new insights into dermoscopic features of lip LP. WS, pigmentation, scales, and telangiectasia are the hallmarks of lip LP. In contrast to the previously reported reticular pattern of cutaneous LP, radial WS are the characteristic feature seen in lip LP. Leaf venation-like WS, hairpin vascular pattern, and rosettes are new dermoscopic features that we did not find described in lip LP.

## Figures and Tables

**Table 1:** Demographic Profile of Patients With Duration of Disease

	Number	Percentage
Gender		
Male	8	66.7%
Female	4	33.3%
Age (years)		
<20	1	8.3%
21–40	2	16.7%
41–60	7	58.3%
>60	2	16.7%
Duration (months)		
<6	2	16.7%
6–12	4	33.3%
12–24	4	33.3%
>24	2	16.7%
Site of lesions		
Lower lip	10	83.3%
Both lips	02	16.7%

**Table 2:** Frequency of Dermoscopic Features in Lip Lichen Planus (n=12)

Study	Dermoscopic Feature	Number	Percentage
01	Wickham striae	12	100%
	Leaf venation-like	02	16.7%
	Radial	09	75%
	Linear	04	33.3%
02	Scales	12	100%
03	Pigmentation	12	100%
	Gray-black granules, globules	10	83.3%
	Brown	02	16.7%
04	Vascular pattern	11	91.7%
	Linear	10	83.3%
	Hairpin	08	66.7%
	Dotted	09	75%
05	Background		
	Erythematous	07	58.3%
	Violaceous	05	41.7%
06	Erosion	06	50%
07	Bleeding spots	04	33.3%
08	Rosettes at lip margin	04	33.3%
